# High-Quality Library Preparation for NGS-Based Immunoglobulin Germline Gene Inference and Repertoire Expression Analysis

**DOI:** 10.3389/fimmu.2019.00660

**Published:** 2019-04-05

**Authors:** Néstor Vázquez Bernat, Martin Corcoran, Uta Hardt, Mateusz Kaduk, Ganesh E. Phad, Marcel Martin, Gunilla B. Karlsson Hedestam

**Affiliations:** ^1^Department of Microbiology, Tumor and Cell Biology, Karolinska Institutet, Stockholm, Sweden; ^2^Division of Rheumatology, Department of Medicine, Center for Molecular Medicine, Karolinska Institutet and Karolinska University Hospital, Stockholm, Sweden; ^3^Science for Life Laboratory, Department of Biochemistry and Biophysics, Stockholm University, Stockholm, Sweden

**Keywords:** next generation sequencing, immunoglobulin, antibody, repertoire, library, germline gene, inference, database

## Abstract

Next generation sequencing (NGS) of immunoglobulin (Ig) repertoires (Rep-seq) enables examination of the adaptive immune system at an unprecedented level. Applications include studies of expressed repertoires, gene usage, somatic hypermutation levels, Ig lineage tracing and identification of genetic variation within the Ig loci through inference methods. All these applications require starting libraries that allow the generation of sequence data with low error rate and optimal representation of the expressed repertoire. Here, we provide detailed protocols for the production of libraries suitable for human Ig germline gene inference and Ig repertoire studies. Various parameters used in the process were tested in order to demonstrate factors that are critical to obtain high quality libraries. We demonstrate an improved 5′RACE technique that reduces the length constraints of Illumina MiSeq based Rep-seq analysis but allows for the acquisition of sequences upstream of Ig V genes, useful for primer design. We then describe a 5′ multiplex method for library preparation, which yields full length V(D)J sequences suitable for genotype identification and novel gene inference. We provide comprehensive sets of primers targeting IGHV, IGKV, and IGLV genes. Using the optimized protocol, we produced IgM, IgG, IgK, and IgL libraries and analyzed them using the germline inference tool IgDiscover to identify expressed germline V alleles. This process additionally uncovered three IGHV, one IGKV, and six IGLV novel alleles in a single individual, which are absent from the IMGT reference database, highlighting the need for further study of Ig genetic variation. The library generation protocols presented here enable a robust means of analyzing expressed Ig repertoires, identifying novel alleles and producing individualized germline gene databases from humans.

## Introduction

The development of NGS-based approaches to Ig repertoire analysis offers new opportunities to investigate B cell responses in health and disease [reviewed in (1–8)]. The Adaptive Immune Receptor Repertoire (AIRR) Community are working actively to develop minimum standards and recommendations for repertoire sequencing studies (9). Commonly utilized approaches to immune repertoire sequencing analysis involve the production of isotype-specific libraries of the Ig cDNA. These libraries are sequenced using NGS protocols that enable the production of amplicons that encompass either partial, in the case of libraries which use framework 1 located primer sequences (10, 11), or full-length sequences of the recombined *variable* (V), *diversity* (D), and *joining* (J) gene segments of Ig heavy chains (HC) or VJ sequences of Ig light chains (LC, kappa or lambda). This requires a sequence length of at least 400 base pairs (bp), thereby limiting the available sequencing platform options. In order to produce libraries of sufficient length and depth for Rep-seq analysis many groups currently utilize long-read Illumina protocols, such as the 2 × 250 bp HiSeq system or, more commonly, the 2 × 300 bp MiSeq system. Two major library production techniques, 5′ Rapid Amplification of cDNA Ends (5′RACE) (12, 13) and 5′ multiplex (5′MTPX) PCR (14–16), are used by researchers working with Rep-seq NGS.

An important first step of Rep-seq analysis, which is required for correct gene assignment and somatic hypermutation (SHM) analysis, is to define the specific germline V alleles present in the subject of interest. The current public database for Ig germline genes, the international ImMunoGeneTics information system (IMGT) (17), includes alleles from a relatively small number of individuals and thus incompletely covers human global diversity. Thus, there is a need for robust library production protocols suitable for germline gene inference that satisfy several critical requirements. First, the library sequence length must be sufficiently short, such that it does not exceed the technical limitations of the sequencing technology used. Second, the library sequences should be of sufficient length such that they include the entire recombined V(D)J sequence internal to the amplification primers. Third, the library amplification should be unbiased to allow inclusion of all V genes utilized in the expressed HC or LC (IgK or IgL) repertoires and represent a high level of diversity of V(D)J sequences.

Careful positioning of constant region primers is the primary means of minimization of amplicon length in Rep-seq library production. Primer localization in the vicinity of the proximal exonic border is commonly used to minimize the overall library sequence length. The 5′ border of the library will be determined by the methodology used, either 5′MTPX primers positioned in the leader or 5′ untranslated region (UTR) of the respective target genes, or a template switch universal amplification sequence added upstream of the 5′UTR during cDNA synthesis (5′RACE). In addition, many current Rep-seq analysis tools take advantage of UMIs added during library production (18–22). UMIs are usually located in the 3′ end of 5′MTPX libraries and at the 5′ end of template-switched 5′RACE libraries (10, 13). The use of UMIs allows the identification of sequences arising from the same mRNA molecule and facilitates error correction of Ig sequences. However, this approach comes at the cost of increasing the sequence length of libraries that strain the currently available high-throughput NGS methods.

Here, we describe the technical limitations of some current library production techniques and we offer methodological solutions to facilitate the use of either 5′RACE or 5′MTPX methods to produce Ig libraries for Rep-seq analysis. In particular, we examine factors that may result in lack of full-length coverage of the expressed Ig repertoire as well as aspects critical to minimize PCR and instrument-associated sequencing errors. The optimized protocols described here facilitate the production of high-quality libraries for germline genotype inference and Ig Rep-seq expression analysis.

## Results

### 5′RACE Library Preparation

**Figure 1:**
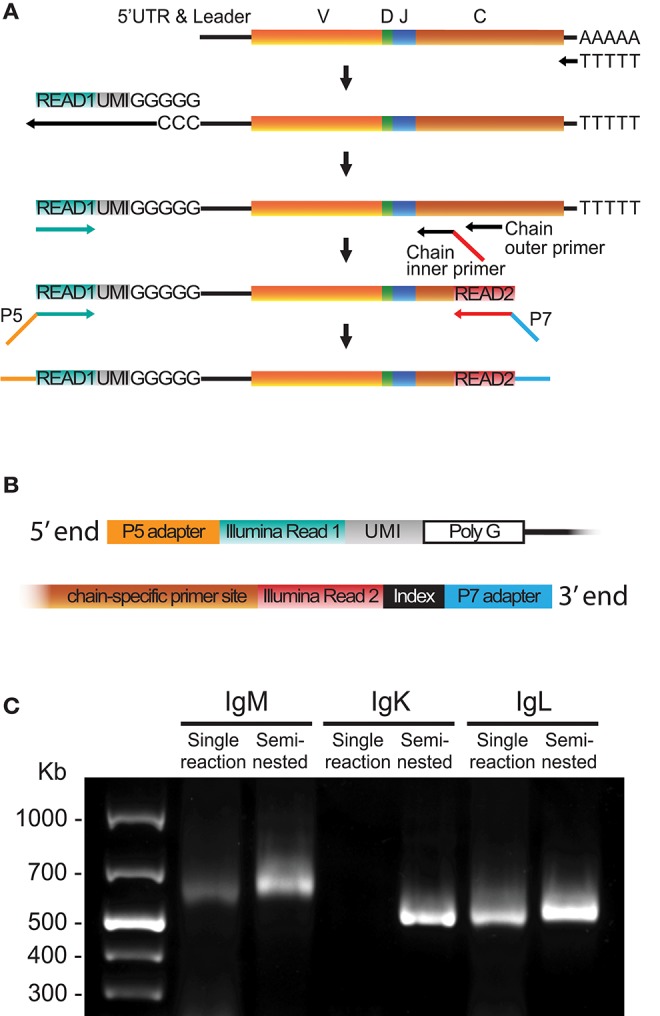
5′RACE library preparation. **(A)** Schematic of the steps required to generate libraries using 5′RACE including cDNA synthesis, template switch reaction, cDNA amplification and addition of Illumina adaptors. **(B)** Schematic of the 5′ and 3′ termini of a final library product produced using the 5′RACE protocol. **(C)** A representative 1% agarose gel comparing library products obtained using single reaction PCR or semi-nested PCR of human IgM, IgK, and IgL cDNA.

To minimize the sequence length for Ig libraries, we developed an optimized 5′RACE cDNA amplification protocol to generate libraries suitable for Illumina MiSeq 2 × 300 bp paired-end sequencing. The procedure is summarized in Figure 1 and in the following steps: First, cDNA synthesis was performed with Oligo-dT and Superscript II, which yields 5′ poly-cytidine overhangs upstream of the UTR. Next, a template switch primer (Read1_TS) containing a poly-guanosine RNA stretch of 5 nucleotides (nt) and a 12 nt UMI and a universal amplification DNA sequence identical to the Illumina's Read1 sequencing primer were added to enable template-switching during the cDNA synthesis step. The purified cDNA template was amplified using a forward Read1-specific primer (Read1U, Figure 1A turquoise) and a reverse chain-specific primer containing a 5′ tail identical to the Illumina Read2 sequence primer (Figure 1A, red). Finally, adapters and indices suitable for Illumina's MiSeq platform were introduced using a 10 cycle PCR step (Figure 1A). The use of a template switch primer identical to the Illumina Read1 reduced the length of the amplicon by 20–25 nt compared to the use of a separate template switch universal amplification sequence. An illustration of the 5′ and 3′ ends of the library amplicon is shown in Figure 1B.

Template switching is a relatively inefficient process in which the yield can be batch-dependent. We found that substitution of a regular 30-cycle cDNA amplification PCR protocol for a semi-nested process increased the yield and lowered the batch variability, especially for the IgM and IgK libraries (Figure 1C). The semi-nested PCR was performed with two consecutive rounds of PCR (20 and 10 cycles, respectively) with inner and outer constant region primers, and the same 5′ Read1U primer. The product from the first PCR was purified and used as template for the second PCR, which introduced the Read2 sequence to the 3′ end of the library (Figure 1A). A protocol describing the 5′RACE library production details is included in Supplementary Material. The 5′RACE libraries include the V gene leader sequence and the 5′ UTR. Acquisition of these sequences provides the bases for the design of gene-specific 5′MTPX primers for full-length V(D)J sequencing. The leader sequences display high conservation between genes of the same IGHV families, making this region optimal for the design of 5′MTPX primers as described below.

### 5′MTPX Library Preparation

**Figure 2:**
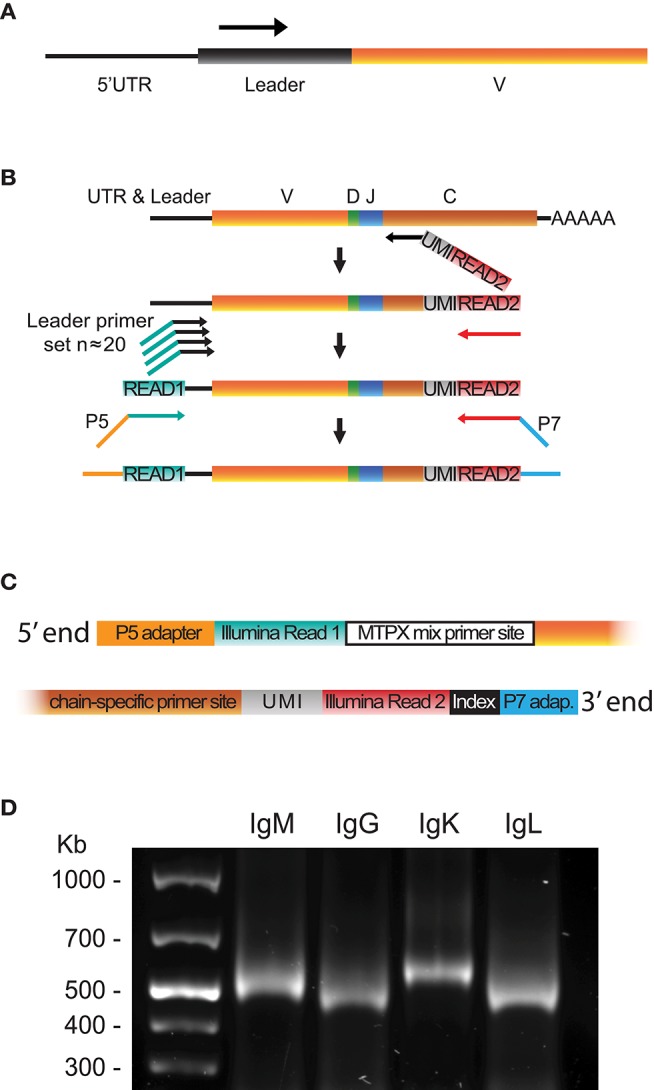
5′MTPX library preparation. **(A)** Position of the 5′MTPX primers in the V gene leader sequence. **(B)** Schematic of the steps required to generate a library product using the 5′MTPX protocol, including cDNA synthesis, cDNA amplification using 5′MTPX primers and addition of Illumina adaptors. **(C)** Schematic of the 5′ and 3′ termini of a final library product produced using the 5′MTPX protocol. **(D)** A representative 1% agarose gel comparing DNA products obtained by 5′MTPX amplification of human IgM, IgG, IgK, and IgL cDNA.

Using the leader sequence information obtained from 5′RACE libraries and available genomic references, we designed sets of 5′MTPX primers positioned in the V gene leader sequences (Supplemental Table 2) for production of full-length human HC and LC V(D)J libraries (Figure 2A). We developed a protocol for 5′MTPX library production summarized in the following steps. First, we performed cDNA synthesis with an IgG, IgM, IgK, or IgL specific 3′ primer that targets the constant regions of the corresponding genes. These gene-specific constant region primers for reverse transcription (RT) also contain a UMI and an adapter tail sequence identical to the Illumina Read2 sequencing primer (Figure 2B, red). Next, we amplified the resulting cDNA template with the 5′MTPX primer mix. Each of the forward primers targeted the leader sequence of one or more V genes and contained a tail identical to the Illumina Read1 sequencing primer (Figure 2B, turquoise). Finally, adapters and indices suitable for Illumina's MiSeq platform were introduced using a 10 cycle PCR step. Figure 2C illustrates the 5′ and 3′ ends of the amplicon produced by the 5′MTPX library production protocol.

Compared to the 5′RACE protocol, the amplicon generated by 5′MTPX does not encompass the poly-cytosine segment, the 5′UTR, and part of the leader sequences, reducing the amplicon length considerably. Thus, the 5′MTPX protocol appeared to have at least two advantages: it involved fewer steps and resulted in shorter amplicons. Furthermore, with the 5′MTPX protocol described here, we obtained comparable yields of library product as when using the 5′RACE protocol, without the need of a semi-nested step and fewer PCR cycles (Figure 2D). A protocol describing the 5′MTPX library production details is included in Supplementary Material.

### Length Constraints in Ig NGS

Illumina's MiSeq 2 × 300 bp V3 kit, commonly utilized in Rep-seq analysis, has a sequencing capability of 600 nt. In theory, this should be sufficient to sequence full-length V(D)J libraries produced by either 5′RACE or 5′MTPX protocols. In practice, however, length variations due to differences in the 5′UTR and/or in the HC complementarity determining region 3 (HCDR3) result in some sequences that are too long for Read1/Read2 merging after 5′RACE library preparation. To merge the Read1 and Read2 sequences, an overlap between the reads of at least 10 nt is required [default of PEAR (23) and FLASH (24)]. This makes the effective read length 2 × 295 nt, including amplification primers and UMI sequences. However, during a typical MiSeq sequencing run, the sequence quality decreases gradually (25), with a significant effect from 275 nt in the Read1 direction and 225 nt in the Read2 direction. Thus, libraries that result in merged sequences above 500 bp in length are associated with higher levels of instrument-based sequence error.

**Figure 3:**
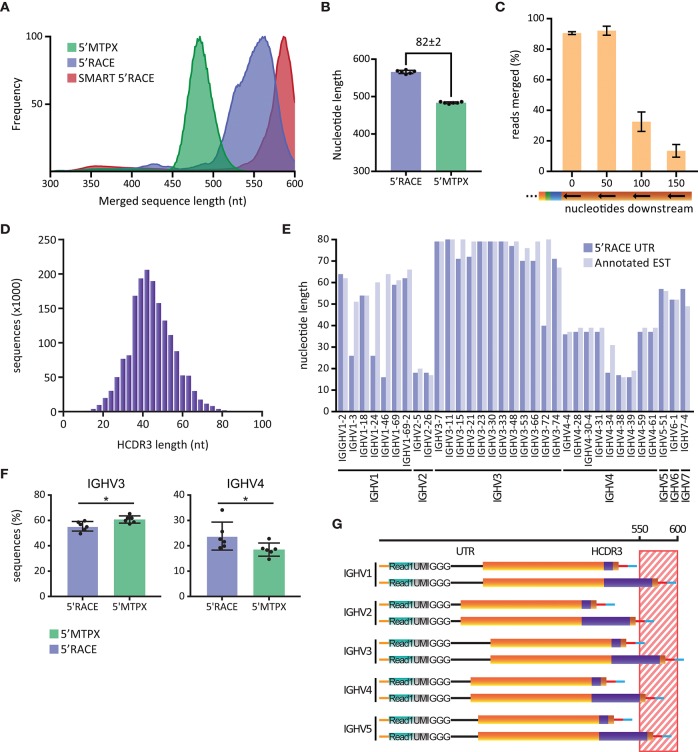
Library length limitations. **(A)** Length distribution of human IgM sequences obtained in one SMART 5′RACE library (red), six 5′RACE libraries (blue) and six 5′MTPX libraries (green), smoothed using 10-point average and normalized. **(B)** Peak of the length distribution of 5′MTPX and 5′RACE in six individuals (mean and SD error bars, m_diff_ = 82 ± 2 nt). **(C)** Percentages of reads merged obtained by IgDiscover analysis of 3 human IgM libraries produced by the 5′MTPX protocol with primers positioned at 0 (original position), 50, 100, or 150 nt downstream in the constant region (mean and SD error bars). **(D)** Distribution of HCDR3 lengths in nt in a human IgM library produced with the 5′MTPX protocol. **(E)** Longest 5′ UTR for different IGHV genes identified in a human IgM library produced by the 5′RACE protocol (dark blue) and the non-unique longest annotated EST (light blue) for each of the genes. **(F)** IGHV3 and IGHV4 gene usage in 5′RACE and 5'MTPX IgM libraries of six individuals (mean and SD error bars, paired t-test, mdiff = 5.4 ± 4.6 and − 5.3 ± 3.8% with ^*^ indicating P < 0.05). **(G)** Schematic (not according to scale) of how 5′ UTR lengths (black line) for different IGHV families, in combination with either a short or a long HCDR3 (purple), contribute to the total length of amplicons generated by 5′RACE. The striped region between 550 and 600 nt indicates sequences that are at risk of being excluded from the analysis due to sequencing length and/or quality limitations.

To investigate the potential impact of this, we produced IgM libraries from six human subjects using either the 5′RACE or the 5′MTPX protocols described above. For comparative purposes, we also generated libraries using the SMART 5′RACE library production. The libraries produced by our 5′RACE protocol were shorter than those produced using the SMART™ 5′RACE protocol, thanks to the primer design described above. The average sequence length of our 5′RACE amplicons was between 550 and 560 nt, while the SMART 5′RACE protocol generated amplicons of 580–600 nt in length (Figure 3A). In contrast, the average sequence length of the amplicons generated by our 5′MTPX protocol was below 500 nt, 82 nt shorter than the amplicons generated by our 5′RACE protocol (Figure 3B).

To investigate how the length differences between the library amplicons impacted the ability to merge Read1 and Read2, we designed a set of alternative 3′ IgM-specific reverse transcription primers for use with the 5′MTPX protocol. The primers were positioned 50, 100, or 150 nt downstream of the position of our standard 3′ primer in the constant region of IgM (Supplemental Table 2). Libraries were prepared from two different individuals using each of the 3′ primers. The libraries were analyzed with IgDiscover (26), which calculates the number of reads that can be merged using the PEAR merger as part of the pre-processing steps. We found that our standard 3′ IgM primer and the 3′ primer positioned 50 nt downstream resulted in 91 and 92% merged reads, respectively. We observed a marked decrease in the number of merged reads for libraries generated with the primers that annealed 100 or 150 nt downstream in the constant region (Figure 3C). Libraries produced with the 3′ primer located 150 nt downstream gave the lowest percentage merged reads. This prevented a complete analysis with IgDiscover, since at least 30% merged reads are required by the program to complete the analysis. This sequence length test indicated that the library length we achieved with the 5′MTPX protocol was >50 nt below the length limit that impacts Read1/2 merging efficiency. In contrast, the two 5′RACE methods, and especially the SMART 5′RACE, resulted in amplicon lengths that were close to the upper limit of what can be accommodated efficiently.

The 5′UTR and CDR3 contribute significantly to length variations of HC VDJ amplicons. To investigate the potential impact of these parameters, we analyzed the range of HCDR3 lengths from a human IgM library produced with the 5′MTPX protocol. Starting from a library of 1.8 million assembled full-length reads, we found HCDR3 lengths in a distribution centered around 42 nt and spanning 12 to 96 nt (Figure 3D). This resulted in a range of 84 nt, which is sufficient to create a potential bias against Ig sequences encoding longer HCDR3s in 5′RACE, or other library production methods that generate amplicons close to the sequencing length limit.

We next examined the lengths of the HC V gene region UTRs. We found that there was a distribution of UTRs with different lengths associated with any given V gene in the libraries. This could be explained by some common limitations of template switching reactions, which result from incomplete reverse transcription of cDNAs. We screened IgM sequences generated by our 5′RACE protocol by inspecting the unmerged Read1 sequences to identify the longest UTR associated with a given IGHV gene. Additionally, we identified the expressed sequence tagged cDNA (EST) associated with the same V gene using the UCSC BLAT genome browser [https://genome.ucsc.edu/ (27)]. We found high consistency in the UTR length from our 5′RACE libraries and the EST for each IGHV gene, with just a few exceptions, such as IGHV1-3, IGHV1-24, IGHV1-46, IGHV3-72, and IGHV4-34 (Figure 3E). We also observed a certain consistency within gene families, with IGHV3 family sequences in general displaying long UTRs and IGHV2 family sequences short UTRs. The difference in length between the longest and the shortest UTRs was centered at 62 nt (18 nt for IGHV2-5 and IGHV2-26 compared to 80 nt for IGHV3-11). When enumerating the number of IGHV3 and IGHV4 sequences in the library data for six individuals, we found that the relative proportion of IGHV3 sequences was lower in the 5′RACE libraries compared to the 5′MTPX libraries, and we observed the opposite for IGHV4 sequences (Figure 3F). These results indicated that IGHV3 amplicons could be lost during merging of the Read1 and Read2 sequences in the 5′RACE libraries. Taken together, these findings suggested that V(D)J transcripts containing the longest UTRs and HCDR3s are at highest risk of being excluded from Read1/Read2 merging of sequences from 5′RACE libraries (Figure 3G). Although 5′RACE should in theory be more unbiased than library production protocols that require gene specific 5′primers, the length differences between different genes may discriminate against longer sequences.

### Quality of Library and Analysis Output

**Figure 4:**
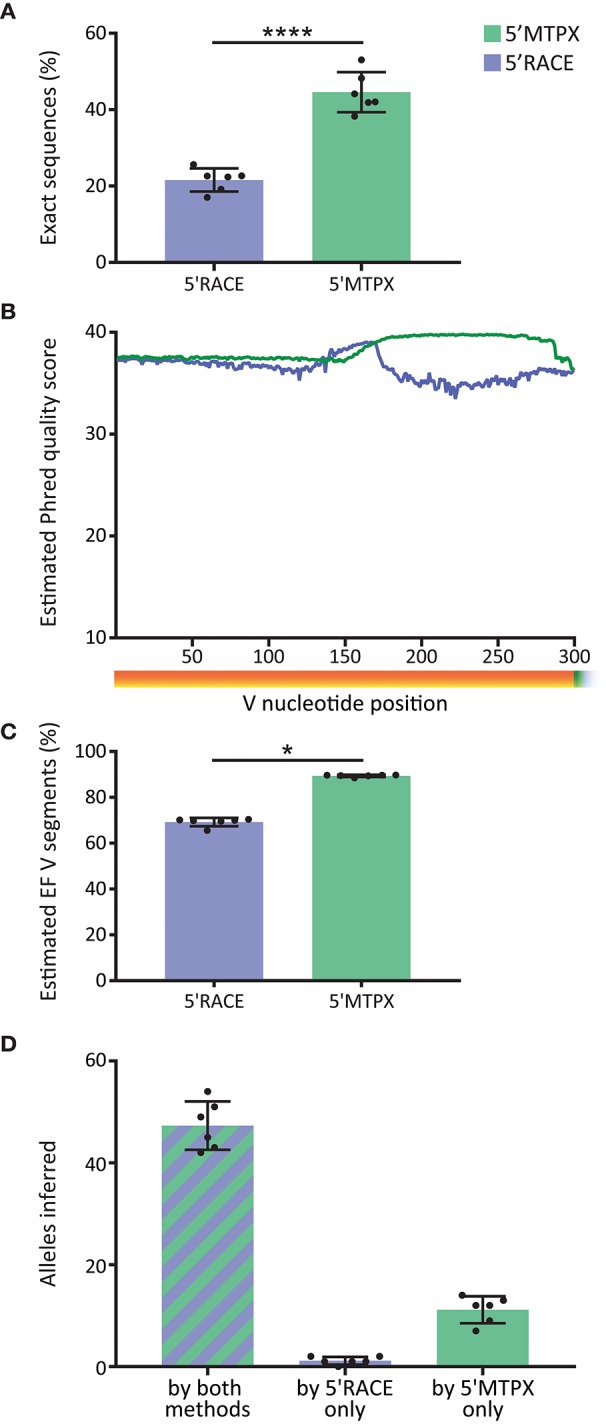
Effect of library production method on sequence quality. **(A)** Percentage of filtered V sequences with 100% identity to reference germline alleles in 5′RACE and 5′MTPX (blue and green, respectively) in the six cindividuals (mean and SD error bars, paired t-test, mdiff = 23 ± 3% with ^****^ indicating P < 0.0001). **(B)** Averaged of the Phred quality score in merged reads in 5′RACE and 5′MTPX in the 300 nt after the beginning of the V gene (six individuals). **(C)** Percentage of sequences expected to have zero sequencing errors in the V segment, inferred from Illumina quality scores (mean and SD error bars, paired Wilcoxon test, median of differences = 19.6 with ^*^ indicating P < 0.05). **(D)** Number of alleles found with IgDiscover per individual with both methods (stripes), only in 5′RACE, or only in 5′MTPX (mean and SD error bars).

Library quality is important for optimal germline gene inference as well as for Rep-seq studies in general. In the IgM libraries produced from the six test subjects, we observed significantly more sequences with V segments that were 100% identical to the inferred germline alleles (“exact sequences”) in the libraries generated by 5′MTPX compared to the libraries generated by 5′RACE (Figure 4A). Those sequences that are not 100% identical to the inferred germline allele consist of a combination of non-naïve IgM with SHM and instrument associated error. The difference between 5′RACE and 5′MTPX is likely due to the length limitations in the Illumina's MiSeq 2 × 300 bp protocol as discussed above, which affect both the merging capacity and the sequence quality. Amplicons above 550 nt include the termini of Read1 and Read2, which have a higher error rate and shorter overlap, thus reducing the confidence of the sequencing base calls. We next investigated the post-merge sequence quality of the reads. The per-base qualities in the fastq reads are an estimate of the chances of a nucleotide error in any given position. The post-merge qualities are calculated by PEAR (23) in IgDiscover, which takes into consideration the overlap. We obtained the Phred quality average of the V segments in 5′MTPX and 5′RACE (Figure 4B), and we observed that although both remained well above Q30 (1 error estimated for every 1,000 nt), 5′RACE had, as expected, a much shorter read overlap (peak around 160 nt), and marked lower quality in the end of the V segment compared to 5′MTPX. For each merged read, we computed the expected number of errors from the quality scores. As the per-base error probabilities are low, we assume that the per-read error is approximately Poisson distributed [Edgar (28)], and used this to compute the probability that each V will be entirely error free (EF). This was then averaged across all reads in each dataset to estimate the expected proportion of EF V segments for that dataset (Figure 4C). This showed again a significant difference between 5′MTPX and 5′RACE, with 19.6% median of difference, similar to the “exact sequence” difference shown in Figure 4A. The post-merged quality score results should be considered as a theoretical estimate since there is an overestimation of sequence quality in the Illumina's Phred score described previously (25, 29, 30). Moreover, the calculation of the merged sequence quality can be overestimated by the merging program (31), and may best be considered a general quality indicator.

Since one objective of Rep-seq analysis is to identify the specific germline alleles expressed in a given individual using germline gene inference tools, we next investigated the output from libraries produced by the 5′RACE or 5′MTPX protocols for the six subjects. Using IgDiscover to generate individualized germline IGHV databases from each donor, we obtained more complete outputs from the libraries generated by the 5′MTPX compared to the 5′RACE protocol. We were able to identify a majority of alleles expressed in each person (mean 47) by both methods. However, we identified on average 11 alleles more per person with the 5′MTPX method (Figure 4B), demonstrating the limitations of the 5′RACE protocol.

In the process of library analysis it is highly beneficial to have indicators of the overall library quality. Using a library with sub-optimal quality limits the ability to generate high-confidence inferred genotypes and can have a direct impact in Ig repertoire expression analysis. IgDiscover, display the numbers of Ds and CDR3s found associated with every allele inferred. These are displayed as “exact Ds” and “exact CDR3s,” which are the number of D alleles and unique HCDR3s associated with sequences that match an inferred allele. We have observed that these numbers are valuable indicators of the overall library quality and diversity. Moreover, polymerases with high fidelity are recommended, as the overall sequence error observed is the addition of the PCR error and the instrument associated error.

**Figure 5:**
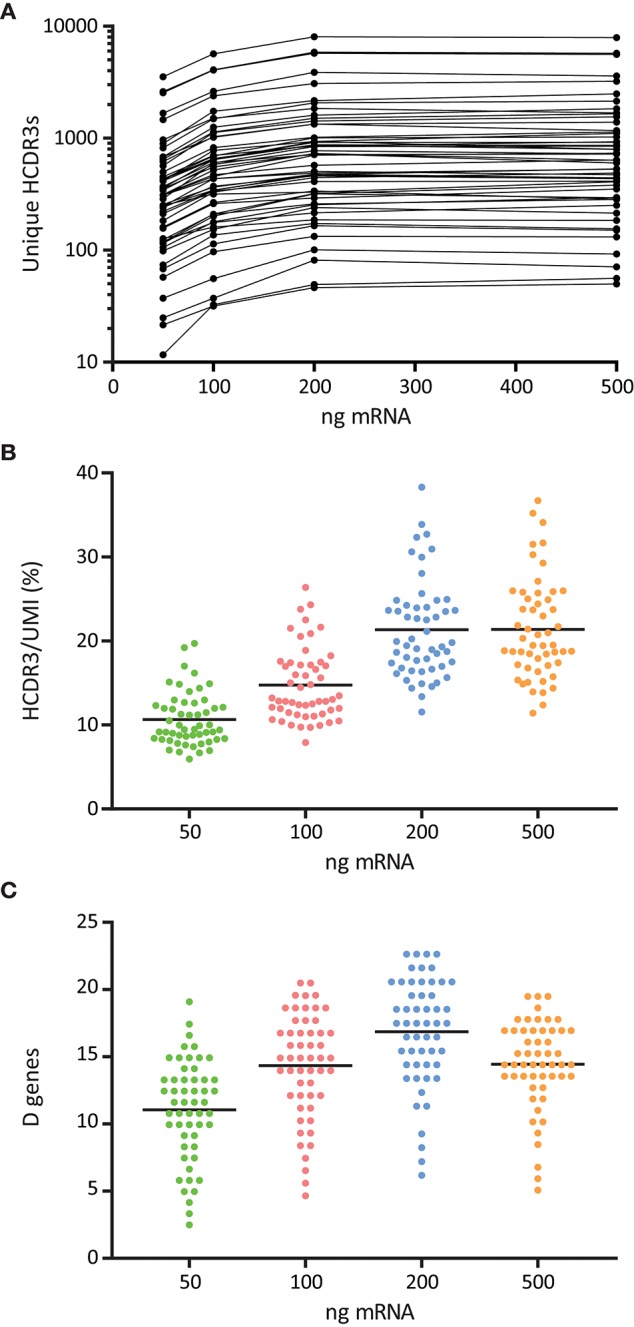
Effect of RNA template amount for the quality and diversity of 5′MTPX libraries. **(A)** Number of unique HCDR3s associated to sequences identical to a given germline V alleles, normalized by library size, shown in logarithmic scale. **(B)** Ratio of unique HCDR3s per unique UMI associated to V sequences identical to a given germline allele. **(C)** Number of unmutated D genes associated to V sequences identical to a given germline allele.

The optimal quantity of RNA required for Ig library production depends on the source of cells used for RNA isolation. In our protocol, we started from total peripheral blood mononuclear cells (PBMCs) where the proportion and composition of B cells is variable (32). We investigated how different starting amounts of PBMC total RNA, namely 50, 100, 200, and 500 ng, affected the quality of the library and the resulting Rep-seq data. We found that libraries generated with 200 and 500 ng RNA were associated with a higher number of unique HCDR3s to each candidate germline V allele (Figures 5A,B). We obtained a similar result when we quantitated the number of unmutated D segments identified per V allele, which suggested increased diversity and quality of libraries generated with 200 ng RNA (Figure 5C).

One concern when preparing libraries for NGS analysis is the generation of PCR artifacts such as chimeric sequences. The number of cycles in the PCR is known to affect chimerism (33). In order to reduce chimerism we used 25 cycles in libraries starting from 200 ng of total PBMC RNA. IgDiscover incorporates a chimera detector for inferred alleles, which flags candidate novel inferred alleles suspected to be a combination of two independent V gene alleles present within that individual's genotype. Although low frequency chimerism was not found to be a major problem for germline gene inference, it may influence lineage tracing or clonal expression analysis, since CDR3s are often used to define clonality.

### Light Chain Library Preparation

**Figure 6:**
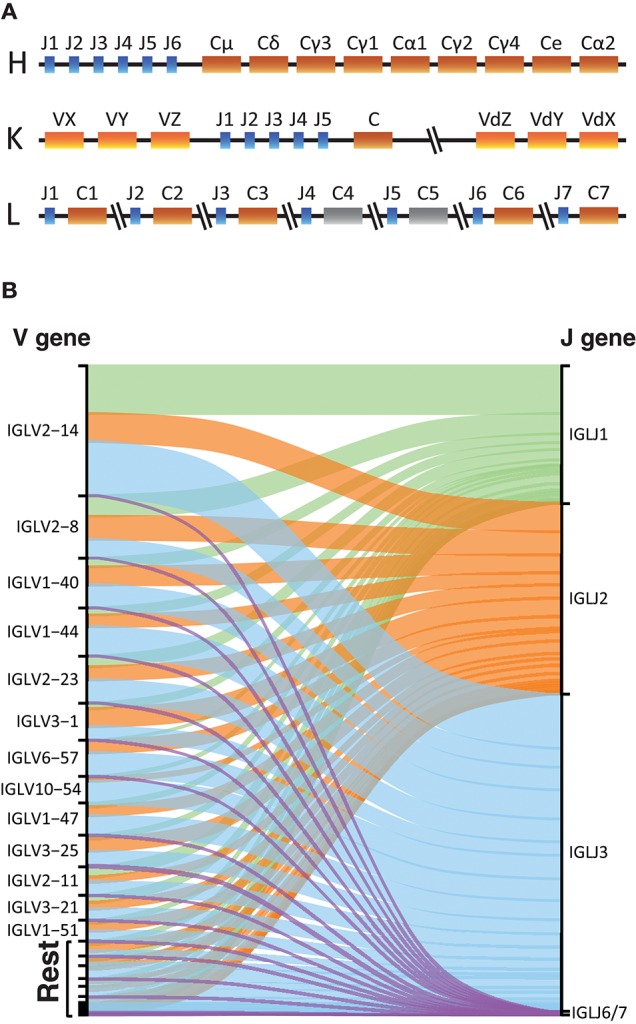
Light chains sequencing. **(A)** Schematic representation of the Heavy, Kappa, and Lambda Ig gene loci in the human genome. In color the functional constant gene segments and in gray the non-functional gene segments. **(B)** Alluvial plot representation of the V-J lambda combinations in the IgL library of one individual with a primer that targets all functional constant genes.

The overall structure of the LC provides advantages and disadvantages for library production and analysis. The lack of D gene segments makes the LC Ig sequences shorter, thus removing the length limitation associated with HC libraries. However, it lowers the variability of the CDR3, which is used to identify unique recombination events during the germline inference process. The organization of the kappa and lambda LC loci differs from that of the HC Ig locus. Ig HCs have different constant regions for different Ig isotypes and subclasses, located downstream of the V, D, and J gene segments. The kappa locus has a single constant gene, but two V gene regions, one upstream of the J genes and one duplicated locus inverted downstream of the constant gene (Figure 6A) (34, 35). Expressed IgK libraries, will contain V genes coming from both IGKV regions. For both the heavy and kappa loci, the gene segments encoding the constant regions are downstream of the J genes. In contrast, the lambda locus encodes 5 functional constant genes interspersed between single lambda J gene in an alternating configuration (Figure 6A) (36).

IgDiscover uses several parameters to identify candidate germline V gene sequences, one of which is the number of J genes associated with a given allele. For IgL library production we found that we could use a single constant region primer that targets a conserved sequence within the genes since we did not see any major difference to the library quality using this method compared to when using a mixture of constant-specific primers (Supplemental Table 2). Furthermore, for IgL repertoires we found that J1, J2, and J3 were the most frequently used genes encompassing the majority of the rearrangements (Figure 6B), consistent with C1, C2, and C3 being the most commonly utilized lambda constant genes.

### Human Libraries and Databases

**Figure 7:**
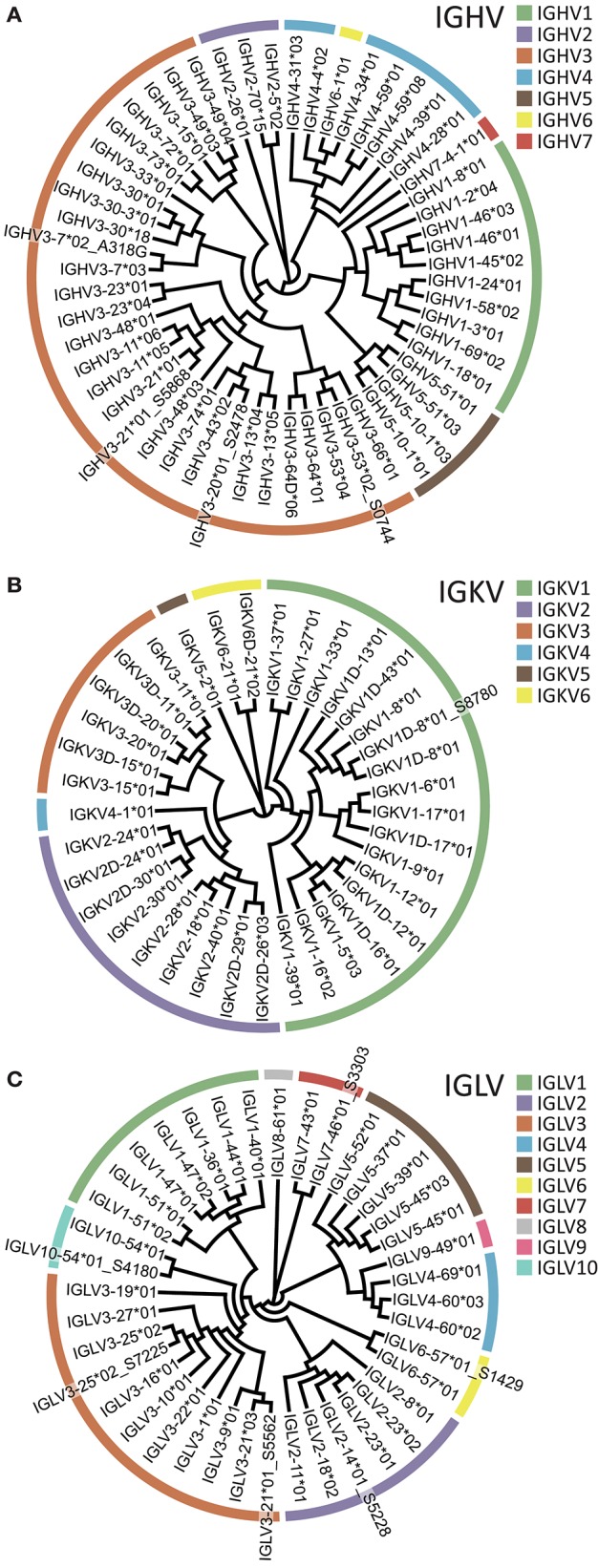
Inferred V alleles in one individual (A007). V gene families are color-coded according to the legend. **(A)** IGHV. **(B)** IGKV. **(C)** IGLV. Novel alleles are denoted using a _S suffix following the name of the closest known reference allele.

Having designed sets of 5′MTPX primers for human heavy and light chain library production (Supplemental Table 2), we sequenced IgM, IgK, and IgL libraries from one individual using the Illumina MiSeq 2 × 300 bp V3 kit. We then used the IgDiscover software (v0.10, Human branch) and constructed individualized germline IGHV, IGKV, and IGLV gene databases. We identified 55 IGHV, 37 IGKV, and 40 IGLV alleles, respectively, in this individual (Figure 7). Of these, we identified several novel alleles so far not described in IMGT: three IGHV alleles, one IGKV allele and six IGLV alleles. One of the novel IGHV alleles, IGLV2-14^*^01_S5228, was identical to IGLV2-14^*^03 (present in IMGT), but with a segment in the 5′ end of 23 nt. We designed primers encompassing the genes of the novel alleles inferred (Supplemental Table 4) and validated all the eleven through genomic DNA amplification, including IGHV3-7^*^02_A318G recently described (37). The novel alleles were expressed at levels comparable to those of known alleles of the same gene, according to the number of UMIs and HCDR3s associated with each allele (Supplemental Figures 1, 2). We also generated an IgG library using a primer designed to match the constant region of IgG1, 2, 3, and 4. Of note, because libraries generated with either the 5′MTPX or the 5′RACE procedure contained UMIs, the analysis of the sequencing results is not restricted to IgDiscover but could also use gene tools such as TIgGER (38). We analyzed the IgM library with TIgGER and identified two out of the three IGHV novel alleles found with IgDiscover (IGHV3-21^*^01_S5868 and IGHV3-53^*^02_S0744). All the libraries and the novel alleles identified in this individual can be found in ENA (accession number ERR3004229-ERR3004232 and ERR3153714-ERR3153725) and Genbank (accession numbers MK308858-MK308867 for the inferred alleles and MK587519-MK587529 for the gDNA validated segments). All the novel alleles will be submitted for consideration to the Inferred All the novel alleles will be submitted for consideration to the Inferred Allele Review Committee (IARC) and IgPdB online database (39).

## Discussion

Rep-seq analysis, in general, involves the assignment of Ig sequences to a reference germline gene database for subsequent identification of Ig expression patterns, SHM and Ig lineages. High fidelity and unbiased library production is a key factor to avoid errors in the subsequent analysis. There is, to date, two main methods for library preparation, but definitive consensus on which is the optimal method has not been reached and this also depends on the study objectives. While 5′MTPX techniques are utilized by many researchers (10, 15), theoretically, the use of a template switch protocol in 5′RACE, which introduces a universal amplification primer site, should give a less biased output since it is sequence independent (40). Our results elucidate pros and cons with the two methods and also indicate additional factors that may significantly impact the library representation.

A critical task in Rep-seq analysis is to ensure that the libraries produced do not exceed the distance capabilities of the available sequencing technologies. Analysis of the variation of 5′ UTR in human heavy chain genes (Figure 3E), shows that the length differs significantly between V genes. For example, IGHV2 family genes have 5′ UTRs with average length of 18 nt while those of the IGHV3 family have significantly longer UTRs with average lengths of 72 nt. This indicates that the use of methods that include the full upstream sequences, such as 5′RACE, may impact our ability to achieve an unbiased representation of the complete repertoire. We show here that the use of a template switch primer containing the same sequence as the Illumina Read1 sequencing primer helps mitigate this issue, by reducing the total amplicon length by 20–25 nt (Figure 3A). This improves the sequencing coverage of libraries generated by 5′RACE but does not eliminate all length constraints, especially for IGHV3 family sequences with long 5′UTRs and long HCDR3s (Figure 3G).

We designed both 5′RACE and 5′MTPX protocols to minimize the amplicon length and facilitate the use of the Illumina-based MiSeq platform. In contrast to alternative longer read approaches, such as PacBio (41), the Illumina MiSeq system results in higher number of individual sequences per run, thereby allowing Rep-seq analysis and germline genotype inference to be performed in a higher throughput manner. The 5′RACE and 5′MTPX approaches described here provide full-length V(D)J sequences suitable for inference of novel V gene alleles, contrary to protocols using primers that target the Ig framework 1 region (11). The identification of an accurate individualized inferred genotype or the detailed analysis of V gene expression requires a suitably unbiased representation of the full range of expressed V genes.

We investigated the amount of RNA used for cDNA synthesis as a factor to ensure high quality library production (Figure 5). These experiments indicated that libraries produced using inadequate amounts of RNA were less diverse and therefore inferior for germline gene inference compared to libraries produced from higher amounts of RNA. This may be important when taking into account the amount of RNA that can be extracted from sorted cells (21).

Moreover, we show how the library quality, as measured by the percentage of sequences identical to assigned germline gene sequences, was higher in libraries generated by 5′MTPX and also yielded improved coverage of the individual's full genotype (Figure 4). Our overall findings suggest that the use of 5′MTPX PCR techniques is more advantageous for MiSeq-based Rep-seq analysis.

There are, however, circumstances where a 5′RACE approach is warranted. Animal models commonly used in research, such as rabbits, guinea pigs, macaques, rats and mice, with the exception of C57BL/6 mice, lack Ig germline gene reference databases suitable for high-quality Rep-seq analysis. Genotype gene inference approaches presents possibilities to construct such Ig gene databases in a short period of time. The 5′RACE protocol described here only requires basic Ig constant region genomic information easily obtainable for many species making this an achievable goal. Moreover, we have shown how the information obtained from 5′RACE libraries can guide the subsequent design of primers for 5′MTPX, which produces libraries with shorter amplicon lengths and hence enables improved sequencing quality. This two-step approach offers opportunities to generate Ig germline gene databases for species where such information is lacking today. In addition to the use of animal models in immunological research, the identification of Ig gene sequence variation in multiple species will be important for understanding evolutionary processes that have shaped the adaptive immune response.

The IMGT database is the most commonly used reference database for Rep-seq analysis; however, it currently lacks coverage of the full range of allelic variation in human populations. Significant allelic differences within the germline V gene repertoire have been observed between individuals and in specific human populations (26, 42–46). Two alleles of a given gene may differ by a single nt or more. The generation of comprehensive reference Ig gene databases therefore requires the identification of as little as single nt mismatches from known germline genes. Furthermore, frequent copy number variations in the HC Ig locus provide additional diversity in humans [Watson (47)]. The identification of the full range of variation within the human Ig germline gene repertoire will require extensive analysis of large numbers of individuals from different populations.

Because genomic sequencing of the Ig loci is challenging and time-consuming, we previously developed a germline inference tool, IgDiscover, which enables the production of individualized Ig germline gene databases from multiple subjects in just a few days (26). Using the optimized 5′MTPX PCR method described in this study, we produced IgM, IgG, IgK, and IgL libraries and we used IgDiscover to identify the full germline V gene genotype for one human volunteer (A007). In addition to known V alleles, we inferred several novel alleles, three IGHV, one IGKV, and six IGLV alleles, demonstrating the value of this approach. If applied to larger sets of individuals, this approach could greatly improve our understanding of human allelic diversity in Ig V genes, as well as help complete the public IMGT database with novel alleles and validation of functional alleles already present the database, thereby ensuring a comprehensive and accurate public database of reference alleles for the research community.

In summary, we describe optimized 5′RACE and 5′MTPX protocols for Ig library production and we compare their use for Rep-seq analysis and individualized germline gene inference, which we believe will be of broad utility for immune repertoire sequencing community.

## Materials and Methods

### Human Subjects and Donor Consent Information

Human PBMCs were obtained from healthy volunteers that signed an informed consent form. All genetic data were acquired digitally and pseudonymized under ethics permits #2016/1053-31 and #2017/852-32 obtained from Regionala Etikprövningsnämnden, Stockholm.

### Isolation and Cryo-Preservation of PBMCs and RNA Extraction

Blood samples were collected in BD Vacutainer® K2 EDTA tubes, and PBMCs were isolated using Ficoll-paque™ (GE Healthcare Life Sciences). The cells were washed twice with PBS, and remaining red cells were removed with lysis buffer. The cells were washed twice again with PBS and frozen at 10 × 10^6^ cells per ml per cryovial in Fetal Bovine Serum (FBS) with 10% DMSO. RNA was extracted using the RNeasy Kit (Qiagen).

### 5′RACE

Two hundred nanogram of total RNA was used for each 5′RACE cDNA synthesis reaction. Superscript II reverse transcriptase (Thermo Scientific) and 10μM Oligo dT was used to generate the first-strand cDNA at 42°C for 1 h. The template switching was performed by adding to the reaction mixture an oligonucleotide containing RNA poly-G sequence, a 12 nucleotide UMI and Illumina's Read1 sequence (Read1_TS in Supplemental Table 1). The addition of the template switch primer was performed at 42°C and the reaction was allowed to proceed for 90 min at 42°C. The cDNA was purified (GeneJET PCR purification Kit, Thermo Scientific) and eluted in 20 μl of TE buffer. 2 μl of the cDNA was amplified in a two-step semi-nested PCR reaction with 3′ chain-specific reverse outer and inner primers, 5′ Read1U forward primer, using KAPA HiFi Hotstart ReadyMix (Kapa Biosystems). The 3′ chain-specific inner primer included a tail with Illumina's Read2 sequence primer (Supplemental Table 1). The PCR products of approximately 550 bp were gel purified using the Qiagen Gel Purification Kit. The primers used were purchased from Integrated DNA Technologies and were purified by standard desalting.

### 5′MTPX

Total RNA (200 ng, unless otherwise mentioned) was used for cDNA synthesis with chain-specific reverse primers (Supplemental Table 2) containing Illumina's Read2 sequence and a UMI for 1 h at 37°C using the Sensiscript RT kit (Qiagen). Two to four microliter of purified cDNA (GeneJET PCR purification Kit, Thermo Scientific) were amplified using the Read2U and the chain-specific 5′ forward leader primer mix (Supplemental Table 2) with the KAPA HiFi Hotstart ReadyMix system (Kapa Biosystems). The PCR product corresponding to the library amplification of approximately 480 bp was gel purified using the Qiagen Gel Purification Kit. The primers used were purchased from Integrated DNA Technologies and were purified by standard desalting.

### NGS Library Production and Sequencing

Five to ten nanogram of the gel-purified amplification product (either 5′MTPX or 5′RACE) was used for index PCR reaction. The forward indexing primer P5_R1 and reverse indexing primer P7_R2_I1-27 were added in a 10 cycle PCR reaction with KAPA HiFi Hotstart ReadyMix system (Kapa Biosystems) (Supplemental Table 3). The primers were purchased from Integrated DNA Technologies and were purified by HPLC. The libraries where purified, validated and quantified according to Illumina's manufacturer's instructions. The Illumina Version 3 (2 x 300 bp) sequencing Kit was used to sequence the libraries with the addition of 13% PhiX174 DNA (12 pM).

### Computational Analysis

The IgDiscover pipeline v0.10 Human branch (https://github.com/mateuszatki/IgDiscover/) was used to pre-process the libraries for quality control, and subsequently perform expression analysis and generate individualized databases. The analysis was performed with the default settings, specifying the pre-CDR3 position ([−135, −80] for IgM 5′RACE, [−145, −90] for IgM 5′MTPX, [−165, −130] for IgK 5′MTPX and [−125, −90] for IgL 5′MTPX), and UMI lengths of 25 nt in 5′RACE and 21 nt in 5′MTPX. The IMGT reference database (April 2018) was used, with the addition of a recently described new allele (37). We used the “igdiscover upstream” subcommand to retrieve the UTR and leader segments of every V allele of the 5′RACE libraries. This subcommand was used with the –max-V-errors = 0 parameter on the filtered.tab.gz file produced by IgDiscover to restrict analysis to sequences without detected sequencing errors in the V segment. 5′MTPX primers were designed on the conserved segments of leader sequences to minimize the total number of primers in the 5′MTPX forward set. CLUSTALW was used for sequence alignment (http://www.genome.jp/tools-bin/clustalw) and BLAT [UCSC genome browser (27)] for the genome confirmations and design of constant region primers. The databases were aligned using MUSCLE [Edgar (48)], maximum likelihood trees were generated with RAxML (49) and the trees were plotted with Dendroscope 3 (50). The alluvial analysis was done with R ggalluvial package of ggplot2.

### Genomic Validation

Primers encompassing the genes of the novel alleles were designed using BLAT [UCSC genome browser (27)] (Supplemental Table 4). Two nanogram of gDNA of A007, obtained with Gentra Puregene Blood Kit (Qiagen), were amplified with the primers at 64°C annealing temperature (30 s), and 72°C elongation (40 s) using KAPA HiFi Hotstart ReadyMix system (Kapa Biosystems). The product was gel purified, ligated to CloneJET pJET 1.2 vector (Thermo Scientific) and XL10-Gold Ultracompetent Cells (Agilent) were transformed with 1 μl of ligation product following manufacturer instructions. After overnight growth on a 100 mg/ml ampicillin LB agarose plates, colonies were screened and positive ones grown in LB medium overnight. Bacterial cultures were purified with the GeneJET Plasmid Miniprep Kit (Thermo Scientific) and Sanger sequenced in both forward and reverse direction (GATC https://www.eurofinsgenomics.eu/en/custom-dna-sequencing/gatc-services/).

## Author Contributions

NV and MC: designed the experiments. NV, UH, GP, and MC: performed the experiments; MM and MK: wrote code for IgDiscover. NV, UH, MC, MK, and GK: analyzed the data. NV, MC, and GK: wrote the paper.

### Conflict of Interest Statement

The authors declare that the research was conducted in the absence of any commercial or financial relationships that could be construed as a potential conflict of interest.

## Abbreviations

NGS: Next Generation Sequencing
Ab: Antibody
Rep-seq: Repertoire sequencing
V: Variable
D: Diversity
J: Joining
UMI: Unique Molecular Identifier
MTPX: multiplex
RACE: Rapid Amplification of cDNA Ends
SHM: Somatic hypermutation.
