# Supplementary material for: High-Quality Library Preparation for NGS-Based Immunoglobulin Germline Gene Inference and Repertoire Expression Analysis
Source: Front Immunol. 2019 Apr 5;10:660. doi: 10.3389/fimmu.2019.00660 (PMC6459949; doi:10.3389/fimmu.2019.00660)
Supplement: Supplementary file 1 [file Table_1.pdf]

Supplemental table 1. Primers used for library preparation in 5’RACE.

**5'RACE**	
**5' primers**	
Read1_TS	CTACACTCTTTCCCTACACGACGCTCTTCCGATCTNNNNNNNNNNNNrGrGrGrGrG
Read1U	CTACACTCTTTCCCTACACGACGCTCTTCCGATCT
**3' primers**	
Hu_IgM_RACE_In	CAGACGTGTGCTCTTCCGATCTGGGAATTCTCACAGGAGACGAGGGGGAAA
Hu_IgK_RACE_In	CGTGTGCTCTTCCGATCTCGGGeAAGATGAAGACAGATGGTGCAGC
Hu_IgL_RACE_In1	GGAGTTCAGACGTGTGCTCTTCCGATCTGAGGAGGGCGGGAACAGAGTGAC
Hu_IgL_RACE_In2	GGAGTTCAGACGTGTGCTCTTCCGATCTCAGAGGAGGGTGGGAACAGAGTGAC
Hu_IgM_RACE_Out	GCCAACGGCCACGCTGCTCGTATCCGA
Hu_IgK_RACE_Out	GGCCTCTCTGGGATAGAAGTTATTCAGCAGGC
Hu_IgL_RACE_Out1	GACACACTAGTGTGGCCTTGTTGGCTTG
Hu_IgL_RACE_Out2	GACACACCAGTGTGGCCTTGTTGGCTTG
Hu_IgL_RACE_Out3	GGCACACCAGTGTGGCCTTGTTGGCTTG
Hu_IgL_RACE_Out4	GACACACCAGCATGGCCTTGTTGGCTTG
