# Supplementary material for: High-Quality Library Preparation for NGS-Based Immunoglobulin Germline Gene Inference and Repertoire Expression Analysis
Source: Front Immunol. 2019 Apr 5;10:660. doi: 10.3389/fimmu.2019.00660 (PMC6459949; doi:10.3389/fimmu.2019.00660)
Supplement: Supplementary file 2 [file Table_2.pdf]

Supplemental table 2. Primers used for library preparation in 5’MTPX.

5'MTPX	
**3' primers**	
Hu_IgM_MTPX	GGAGTTCAGACGTGTGCTCTTCCGATCTHHHHHACAHHHHHACAHHHHNHCCGACGGGGAATTCTCACAGGAGACGAGGGGGAAAAG
Hu_IgM_MTPX-50	GGAGTTCAGACGTGTGCTCTTCCGATCTHHHHHACAHHHHHACAHHHHGAGTTGTTCTTGTATTTCCAGGAGAAAGTGATGGA
Hu_IgM_MTPX-100	GGAGTTCAGACGTGTGCTCTTCCGATCTHHHHHACAHHHHHACAHHHHCCCTCTCAGGACTGATGGGAAGC
Hu_IgM_MTPX-150	GGAGTTCAGACGTGTGCTCTTCCGATCTHHHHHACAHHHHHACAHHHHCGTCCTTGGAAGGCAGCAGCACC
Hu_IgG_MTPX	GGAGTTCAGACGTGTGCTCTTCCGATCTHHHHHACAHHHHHACAHHHHGCCAGGGGGAAGACCGATGGG
Hu_IgK_MTPX	GGAGTTCAGACGTGTGCTCTTCCGATCTHHHHHACAHHHHHACAHHHHNGGGATAGAAGTTATTCAGCAGGCACACAACAGAG
Hu_IgL_MTPX	GGAGTTCAGACGTGTGCTCTTCCGATCTHHHHHACAHHHHHACAHHHHTGGCTTGRAGCTCCTCAGAGGAGG
Read2U	GGAGTTCAGACGTGTGCTCTTCCGATCT
**5' primers**	
Hu_VH_MTPX_1	CCTACACGACGCTCTTCCGATCTGGTGGCAGCAGTCACAGATGCCTACTC
Hu_VH_MTPX_2	CCTACACGACGCTCTTCCGATCTGGTGGCAGCAGCCACAGGTGCCCACTC
Hu_VH_MTPX_3	CCTACACGACGCTCTTCCGATCTGGTGGCAGCAGCTACAGGTGTCCAGTC
Hu_VH_MTPX_4	CCTACACGACGCTCTTCCGATCTGGTGGSAGCAGCAACARGWGCCCACTC
Hu_VH_MTPX_5	CCTACACGACGCTCTTCCGATCTGCTGGCTGTAGCTCCAGGTGCTCACTC
Hu_VH_MTPX_6	CCTACACGACGCTCTTCCGATCTCCTGCTGCTGACCAYCCCTTCMTGGGTCTTGTC
Hu_VH_MTPX_7	CCTACACGACGCTCTTCCGATCTCCTGCTACTGACTGTCCCGTCCTGGGTCTTATC
Hu_VH_MTPX_8	CCTACACGACGCTCTTCCGATCTGGGTTTTCCTCGTTGCTCTTTTAAGAGGTGTCCAGTG
Hu_VH_MTPX_9	CCTACACGACGCTCTTCCGATCTGGGTTTTCCTTGTTGCTATTTTAAAAGGTGTCCARTG
Hu_VH_MTPX_10	CCTACACGACGCTCTTCCGATCTGGATTTTCCTTGCTGCTATTTTAAAAGGTGTCCAGTG
Hu_VH_MTPX_11	CCTACACGACGCTCTTCCGATCTGGGTTTTCCTTKTKGCTATWTTAGAAGGTGTCCAGTG
Hu_VH_MTPX_12	CCTACACGACGCTCTTCCGATCTGGTGGCRGCTCCCAGATGGGTCCTGTC
Hu_VH_MTPX_13	CCTACACGACGCTCTTCCGATCTCTGGCTGTTCTCCAAGGAGTCTGTG
Hu_VH_MTPX_14	CCTACACGACGCTCTTCCGATCTGGCCTCCCATGGGGTGTCCTGTC
Hu_VH_MTPX_15	CCTACACGACGCTCTTCCGATCTGGTGGCAGCAGCAACAGGTGCCCACT
Hu_VH_MTPX_16	CACTCTTTCCCTACACGACGCTCTTCCGATCTATGGAACTGGGGCTCCGCTGGGTTTTCC
Hu_VH_MTPX_17	CACTCTTTCCCTACACGACGCTCTTCCGATCTATGGACTGCACCTGGAGGATCCTCCTC
Hu_VH_MTPX_18	CACTCTTTCCCTACACGACGCTCTTCCGATCTTGGCTGAGCTGGGTTTYCCTTGTTGC
Hu_VH_MTPX_19	CACTCTTTCCCTACACGACGCTCTTCCGATCTGGAGTTKGGGCTGMGCTGGGTTTTCC
Hu_VH_MTPX_20	CACTCTTTCCCTACACGACGCTCTTCCGATCTGCACCTGTGGTTTTTCCTCCTGCTGGTG
Hu_VH_MTPX_21	CACTCTTTCCCTACACGACGCTCTTCCGATCTCACCTGTGGTTCTTCCTCCTSCTGG
Hu_VH_MTPX_22	CACTCTTTCCCTACACGACGCTCTTCCGATCTCCAGGATGGGGTCAACCGCCATCCTC
Hu_VH_MTPX_23	CTCTTTCCCTACACGACGCTCTTCCGATCTCAGAGGACTCACCATGGAGTTTGGGCTGAG
Hu_VH_MTPX_24	CCTACACGACGCTCTTCCGATCTGGACTCACCATGGAGTTGGGACTGAGC
Hu_VH_MTPX_25	CCTACACGACGCTCTTCCGATCTGGGCTGAGCTGGCTTTTTCTTGTGGC
Hu_VK_MTPX_1	CTACACTCTTTCCCTACACGACGCTCTTCCGATCTATGTTGCCATCACAACTCATTGGGTTTCTG
Hu_VK_MTPX_2	CTACACTCTTTCCCTACACGACGCTCTTCCGATCTATGGAARCCCCAGCGCAGCTTCTCTTCC
Hu_VK_MTPX_3	CTACACTCTTTCCCTACACGACGCTCTTCCGATCTATGAGGCTCCCTGCTCAGCTCTTGGGGCT
Hu_VK_MTPX_4	CTACACTCTTTCCCTACACGACGCTCTTCCGATCTATGAGGCTCCCTGCTCAGCTCCTGGGGCT
Hu_VK_MTPX_5	CTACACTCTTTCCCTACACGACGCTCTTCCGATCTATGGACATGAGGGTCCCTGCTCAGC
Hu_VK_MTPX_6	CTACACTCTTTCCCTACACGACGCTCTTCCGATCTATGGACATGAGRGTCCTCGCTCAGC
Hu_VK_MTPX_7	CTACACTCTTTCCCTACACGACGCTCTTCCGATCTATGGAAGCCCCAGCACAGCTTCTTCTTCC
Hu_VK_MTPX_8	CTACACTCTTTCCCTACACGACGCTCTTCCGATCTATGAGGCTCCTTGCTCAGCTTCTGGGGCT
Hu_VK_MTPX_9	CTACACTCTTTCCCTACACGACGCTCTTCCGATCTATGGAAGCCCCAGCTCAGCTTCTCTTCC
Hu_VK_MTPX_10	CTACACTCTTTCCCTACACGACGCTCTTCCGATCTATGGACATGAGGGTCCCCGCTCAGC
Hu_VK_MTPX_11	CTACACTCTTTCCCTACACGACGCTCTTCCGATCTATGGGGTCCCAGGTTCACCTCCTCAG
Hu_VK_MTPX_12	CTACACTCTTTCCCTACACGACGCTCTTCCGATCTATGGTGTTGCAGACCCAGGTCTTCATTTC

Hu_VK_MTPX_13	CTACACTCTTTCCCTACACGACGCTCTTCCGATCTATGGACATGAGGGTGCCCGCTCAGC
Hu_VK_MTPX_14	CTCTTTCCCTACACGACGCTCTTCCGATCTCAGGAAGATGTYGCCATCACAACTCATTGG
Hu_VK_MTPX_15	CACTCTTTCCCTACACGACGCTCTTCCGATCTCTCRCAATGAGGCTCCCTGCTCAGCTC
Hu_VK_MTPX_16	CACTCTTTCCCTACACGACGCTCTTCCGATCTCCTGCTCAGCTCYTGGGGCTGCTAATGC
Hu_VK_MTPX_17	CACTCTTTCCCTACACGACGCTCTTCCGATCTATGGACATGAGGGTGCCCGCTCAGCGCC
Hu_VK_MTPX_18	CACTCTTTCCCTACACGACGCTCTTCCGATCTATGGACATGAGGGTSCCYGCTCAGCKCC
Hu_VK_MTPX_19	CACTCTTTCCCTACACGACGCTCTTCCGATCTGCTCCTGGGGCTGCTAATGCTCTGG
Hu_VK_MTPX_20	CACTCTTTCCCTACACGACGCTCTTCCGATCTGGGGCTCCTGCTGCTCTGGCTCC
Hu_VK_MTPX_21	CACTCTTTCCCTACACGACGCTCTTCCGATCTGGACATGAGGGTCCCCGCTCAGCTCC
Hu_VL_MTPX_1	CTACACTCTTTCCCTACACGACGCTCTTCCGATCTATGGCCTGGGCTCCACTACTTCTCACCCTCC
Hu_VL_MTPX_2	CTACACTCTTTCCCTACACGACGCTCTTCCGATCTATGGCCTGGTCCCCTCTCTTCCTCACCCT
Hu_VL_MTPX_3	CTACACTCTTTCCCTACACGACGCTCTTCCGATCTATGGCCTGGGCTCTGCTCCTCCTCACCCT
Hu_VL_MTPX_4	CTACACTCTTTCCCTACACGACGCTCTTCCGATCTATGGCCTGGAYCCCTCTCCTGCTCCCCCTC
Hu_VL_MTPX_5	CTACACTCTTTCCCTACACGACGCTCTTCCGATCTATGGCCTGGGCTCTGCTGCTCCTCACTCT
Hu_VL_MTPX_6	CTACACTCTTTCCCTACACGACGCTCTTCCGATCTATGGCATGGATCCCTCTCTTCCTCGGCGTC
Hu_VL_MTPX_7	CTACACTCTTTCCCTACACGACGCTCTTCCGATCTATGGCATGGGCCACACTCCTGCTCCCACTC
Hu_VL_MTPX_8	CTACACTCTTTCCCTACACGACGCTCTTCCGATCTATGGCCTGGGTCTCCTTCTACCTACTGCCCT
Hu_VL_MTPX_9	CTACACTCTTTCCCTACACGACGCTCTTCCGATCTATGGCCTGGACTCCTCTTCTTCTCTTGCTCCT
Hu_VL_MTPX_10	CTACACTCTTTCCCTACACGACGCTCTTCCGATCTATGGCCTGGACTCCTCTCCTCCTCCTGYTCC
Hu_VL_MTPX_11	CTACACTCTTTCCCTACACGACGCTCTTCCGATCTATGAGTGTCCCCACCATGGCCTGGATGATGC
Hu_VL_MTPX_12	CTACACTCTTTCCCTACACGACGCTCTTCCGATCTATGGCCTGGGCTCCTCTGCTCCTCACCCTCC
Hu_VL_MTPX_13	CTACACTCTTTCCCTACACGACGCTCTTCCGATCTATGRCCDGCTTCCCTCTCCTCCTCACCCT
Hu_VL_MTPX_14	CTACACTCTTTCCCTACACGACGCTCTTCCGATCTATGGCCTGGACCCCACTCCTCCTCCTCTTCC
Hu_VL_MTPX_15	CTACACTCTTTCCCTACACGACGCTCTTCCGATCTATGGCCTGGGCTCTGCTSCTCCTCASCCT
Hu_VL_MTPX_16	CTACACTCTTTCCCTACACGACGCTCTTCCGATCTATGGCCTGGATCCCTCTACTTCTCCCCCTC
Hu_VL_MTPX_17	CTACACTCTTTCCCTACACGACGCTCTTCCGATCTATGGCCTGGACCSCTCTCCTCCTCRGCCTC
Hu_VL_MTPX_18	CTACACTCTTTCCCTACACGACGCTCTTCCGATCTATGGCCTGGACTCTTCTCCTTCTCGTGCTCC
Hu_VL_MTPX_19	CTACACTCTTTCCCTACACGACGCTCTTCCGATCTATGGCCTGGTCTCCTCTCCTCCTCACTCT
Hu_VL_MTPX_20	CTACACTCTTTCCCTACACGACGCTCTTCCGATCTATGCCCTGGGCTCTGCTCCTCCTGACCCT
Hu_VL_MTPX_21	CTACACTCTTTCCCTACACGACGCTCTTCCGATCTATGGCCTGGACCCCTCTCTGGCTCACTCTC
Hu_VL_MTPX_22	CTACACTCTTTCCCTACACGACGCTCTTCCGATCTATGGCCTGGACCGCTCTCCTTCTGAGCCTC
Hu_VL_MTPX_23	CTACACTCTTTCCCTACACGACGCTCTTCCGATCTATGGCTTGGACCCCACTCCTCTTCCTCACC
Hu_VL_MTPX_24	CTACACTCTTTCCCTACACGACGCTCTTCCGATCTATGGCCTGGACTCCTCTCTTTCTGTTCCTCC
Hu_VL_MTPX_25	CACTCTTTCCCTACACGACGCTCTTCCGATCTATGGCCTGGACTCTTCTCCTTCTCGTG
Hu_VL_MTPX_26	CACTCTTTCCCTACACGACGCTCTTCCGATCTATGGCCTGGACTCCTCTYCTYCTCYTG
Hu_VL_MTPX_27	CACTCTTTCCCTACACGACGCTCTTCCGATCTATGGCCTGGACCCCACTCCTCCTC
Hu_VL_MTPX_28	CACTCTTTCCCTACACGACGCTCTTCCGATCTATGGCCTGGGTCTCCTTCTACCTACTGC
Hu_VL_MTPX_29	CACTCTTTCCCTACACGACGCTCTTCCGATCTGCAGCATCGGAGGTGCCTCAGCCATG
Hu_VL_MTPX_30	CACTCTTTCCCTACACGACGCTCTTCCGATCTGGCAGAACTCTGGGTGTCTCACCATG
Hu_VL_MTPX_31	CACTCTTTCCCTACACGACGCTCTTCCGATCTGCAGCACTGGTGGTGCCTCAGCCATG
Hu_VL_MTPX_32	CACTCTTTCCCTACACGACGCTCTTCCGATCTGGGCTCTGCTSCTCCTCACYCTCCT
Hu_VL_MTPX_33	CACTCTTTCCCTACACGACGCTCTTCCGATCTGGGCTCTGCTCCTCCTGACCCTC
