# Supplementary material for: High-Quality Library Preparation for NGS-Based Immunoglobulin Germline Gene Inference and Repertoire Expression Analysis
Source: Front Immunol. 2019 Apr 5;10:660. doi: 10.3389/fimmu.2019.00660 (PMC6459949; doi:10.3389/fimmu.2019.00660)
Supplement: Supplementary file 4 [file Table_4.pdf]

Supplemental table 4. Primers used for genomic DNA validation.

Genomic validation primers	
IGHV3-21R	CGCCGCAGGCCATGACAGGAAGC
IGHV3-21F	CAGCGTCCCACCCTAGAGCTTGT
IGHV3-20_R	GCACCTGGTCCCTGAGTTTACTGTGTTC
IGHV3-20_F	CACGGGCCAGACAGTGAGACTGG
IGHV3-7_R	CCTGGGGAAATTTGACGACGAGGCA
IGHV3-7_F	GGGTACAGCCTATTCCTCCAGCA
IGHV3-53_R	GAGAGGTTCCCTGGGGAACCGT
IGHV3-53_F	CTCCTGTAAAATGTAACATTGAAACCTGCCT
IGK1D-8*01_F	ACTCCGTCGACTCCTGAGGA
IGK1D-8*01_R	GTGTGGCTGCATCTGAGGAACAC
IGLV7-46_F	GAGCAGCTGCTTCCTCCCACAGGAC
IGLV7-46_R	CCCCTTTCTATGGCTGGTCCAC
IGLV6-57_F	CCCAGATCCTCTGCTGTTTTCCCTC
IGLV6-57_R	GGGTAGAGGCCAGAGATGCAAGTC
IGLV2-14_F	CAGCTGAGCTCCACTATCCAAGGAAGC
IGLV2-14_R	GGCCGCACTGCAGGAAGCCGCTTGC
IGLV3-21_F	GAGCCCAGACCATGGGACCAGTG
IGLV3-21_R	GGGCTGGAGGGAAGGGCAACCTG
IGLV3-25_F	CTCCAAGAGGAGTCCCAGAGGAAG
IGLV3-25_R	GGGTGAATTTGGCCAAGCCTACTC
IGLV10-54_F	CCTCTCTGCTTCAGATCTTTGAGTAACCAGCAC
IGLV10-54_R	CAGTGCTCTGTGAGGCCTGTTCAG
