# Supplementary material for: High-Quality Library Preparation for NGS-Based Immunoglobulin Germline Gene Inference and Repertoire Expression Analysis
Source: Front Immunol. 2019 Apr 5;10:660. doi: 10.3389/fimmu.2019.00660 (PMC6459949; doi:10.3389/fimmu.2019.00660)
Supplement: Supplementary file 5 [file Presentation_1.pdf]

Supplementary Material

High-Quality Library Preparation for NGS-Based Immunoglobulin Germline Gene Inference and Repertoire Expression Analysis

Néstor Vázquez Bernat[1], Martin Corcoran[1], Uta Hardt[1,2], Mateusz Kaduk[1], Ganesh E. Phad[1], Marcel Martin[3] and Gunilla B. Karlsson Hedestam[1,*]

*** Correspondence:**
Gunilla B. Karlsson Hedestam, Department of Microbiology, Tumor and Cell Biology, Karolinska Institutet, Box 280, S-171 77 Stockholm, Sweden. E-mail: Gunilla.Karlsson.Hedestam@ki.se

1 Supplementary Protocols

1.1 5'RACE immunoglobulin library preparation protocol

1. Extract the RNA from PBMC using Qiagen's RNeasy® Mini Kit (cat. no. 74104).
2. Determine RNA concentration obtained and use 200 ng in subsequent library preparations.
3. cDNA synthesis with Thermo Scientific's SuperScript™ II Reverse Transcriptase (cat. no. 18064014):

Reaction mix:

- 200 ng RNA (X µl)
- 11-X µl nuclease free H_2O
- 1 µl 10 mM Oligo-dt
- 1 µl 10 mM dNTPs

Incubate for 5 min at 72°C and then bring it down to 4°C.

Add:

- 4 µl 5X FS buffer
- 1 µl 0.1 M DTT
- 1 µl RiboBlock RNAse inhibitor
- 1 µl Superscript II

Incubate for 1 h at 42°C.

Add (inside the PCR machine)**:**

- 1 µl 10 µM Read1_TS
- 0.5 µl Superscript II
- 0.5 µl 5X FS buffer

Incubate for 1 h and 30 min at 42°C, 10 min at 72°C and then bring it down again to 4°C.

4. Purify the cDNA synthesis product with Thermo Scientific's GeneJET PCR Purification Kit (cat. no. K0701) (elute with 25 µl of EB).
5. cDNA amplification PCR:

Semi-Nested 1 mix:

- 2 µl cDNA
- 6.5 µl nuclease free H_2O
- 0.5 µl 10 mM 5' Read1U primer
- 1 µl 10 mM 3' chain-specific outer primer
- 10 µl KAPA mix

PCR protocol:

- 96°C 5 min
- 95°C 20 sec
- 69°C 20 sec
- 72°C 20 sec
- 72°C 5 min

(95°C 20 sec, 69°C 20 sec, 72°C 20 sec: 20 cycles)

→ PCR purification (elute in 10 µl) →

Semi-Nested 2 mix:

- 10 µl PCR 1 product
- 0.5 µl 10 mM 5' Read1U primer
- 1 µl 10 mM 3' chain-specific inner primer
- 11.5 µl KAPA mix

PCR protocol:

- 96°C 5 min
- 95°C 20 sec
- 69°C 20 sec
- 72°C 20 sec
- 72°C 5 mix

(95°C 20 sec, 69°C 20 sec, 72°C 20 sec: 10 cycles)

KAPA mix: Kapa Biosystems' KAPA HiFi HotStart ReadyMix PCR Kit (cat. no. KR0370)

6. Run the PCR product in a 1% Agarose gel in TAE and cut the band.
7. Purify the gel band with the Thermo Scientific's GeneJET Gel Extraction Kit (cat. no. K0692) and (elute with 25 µl of EB).
8. Determine concentration obtained.
9. Index PCR:

Initial mix:

- 2 µl 10 mM P5_R1 (Read1U with P5)
- 2 µl 10 mM P7_R2 (Read2U with index and P7)
- 10 ng purified PCR product (X µl)
- 21-X µl nuclease free H_2O
- 25 µl Kapa Biosystems' KAPA HiFi HotStart ReadyMix PCR Kit (cat. no. KR0370)

PCR protocol:

- 96°C 5 min
- 95°C 30 seconds
- 68°C 30 seconds
- 72°C 30 seconds
- 72°C 10 min

(95°C 30 seconds, 68°C 30 seconds, 72°C 30 seconds: 10 cycles)

10. Purify PCR product using Qiagen's MinElute PCR Purification Kit (cat. no. 28006) (elute with 50 µl of DNAse and RNAse free H_2O H_2O) and proceed to Illumina's bead purification.

1.2 5’MTPX i mMunoglobulin library preparation protocol

1. Extract the RNA from PBMC using Qiagen’s RNeasy® Mini Kit (cat. no. 74104).
2. Determine RNA concentration obtained and use 200 ng in subsequent procedure.
3. cDNA synthesis with Qiagen’s Sensiscript RT Kit (cat. no. 205211):

Reaction mix:

- 200 ng RNA (X µl)
- 12-X µl nuclease free H_2O
- 2 µl 10 mM Class specific primer (with UMI and Read2U sequence)
- 2 µl dNTP Mix (5 mM each dNTP)

Incubate for 5 min at 65°C, 5 min at 37°C and then place the reaction mixtures on ice.

Add from a pre-mix:

- 2 µl 10xbuffer RT
- 1 µl ¼ RiboBlock RNAse inhibitor
- 1 µl Sensiscript RT

Incubate for 1 h at 37°C, then 10 min at 72°C and then bring it down again to 4°C.

4. Purify the cDNA synthesis product with Thermo Scientific’s GeneJET PCR Purification Kit (cat. no. K0701) (elute with 20 µl of EB).
5. cDNA amplification PCR:

Reaction mix:

- 2 µl cDNA (4 µl if necessary)
- 5 µl nuclease free H_2O
- 1 µl 10 mM 3’ Read2U
- 2 µl 1/10 0.1 M 5’ Forward mixture (with Read1U sequence)
- 10 µl Kapa Biosystems’ KAPA HiFi HotStart ReadyMix PCR Kit (cat. no. KR0370)

PCR protocol:

- 96°C 5 min
- 95°C 20 sec } 25 cycles
- 68°C 20 sec } 25 cycles
- 72°C 20 sec } 25 cycles
- 72°C 5 min

6. Run the PCR product in a 1% Agarose gel in TAE and cut the band.
7. Purify the gel band with the Thermo Scientific’s GeneJET Gel Extraction Kit (cat. no. K0692) and (elute with 25 µl of EB).
8. Determine concentration obtained.

9. Index PCR:

Initial mix:

- 2 µl 10 mM P5_R1 (Read1U with P5)
- 2 µl 10 mM P7_R2 (Read2U with index and P7)
- 5-10 ng purified PCR product (X µl)
- 21-X µl nuclease free H_2O
- 25 µl Kapa Biosystems' KAPA HiFi HotStart ReadyMix PCR Kit (cat. no. KR0370)

PCR protocol:

- 96°C 5 min
- 95°C 30 sec } 10 cycles
- 68°C 30 sec } 10 cycles
- 72°C 30 sec } 10 cycles
- 72°C 10 min

10. Purify PCR product using Qiagen's MinElute PCR Purification Kit (cat. no. 28006) (elute with 50 µl of DNAse and RNAse free H2O) and proceed to Illumina's bead purification.
